# Effect of Automated Oxygen Titration during Walking on Dyspnea and Endurance in Chronic Hypoxemic Patients with COPD: A Randomized Crossover Trial

**DOI:** 10.3390/jcm10214820

**Published:** 2021-10-20

**Authors:** Linette Marie Kofod, Elisabeth Westerdahl, Morten Tange Kristensen, Barbara Cristina Brocki, Thomas Ringbæk, Ejvind Frausing Hansen

**Affiliations:** 1 PMR-C, Department of Physio- and Occupational Therapy, Copenhagen University Hospital, 2650 Hvidovre, Denmark; morten.tange.kristensen@regionh.dk; 2 School of Medical Sciences, Faculty of Medicine and Health, Örebro University, 702 81 Örebro, Sweden; 3 University Health Care Research Center, Faculty of Medicine and Health, Örebro University, 702 81 Örebro, Sweden; elisabeth.westerdahl@regionorebrolan.se; 4 Department of Orthopaedic Surgery, Copenhagen University Hospital, Hvidovre, 2650 Hvidovre, Denmark; 5 Department of Physio- and Occupational Therapy, Copenhagen University Hospital, Bispebjerg- Frederiksberg and Department of Clinical Medicine, University of Copenhagen, 2400 Copenhagen, Denmark; 6 Department of Physiotherapy and Occupational Therapy, Aalborg University Hospital, 9000 Aalborg, Denmark; bcb@rn.dk; 7 Department of Pulmonology, Copenhagen University Hospital, Hvidovre, 2650 Hvidovre, Denmark; thomasringbaek@gmail.com (T.R.); ejvind.frausing.hansen@regionh.dk (E.F.H.)

**Keywords:** physiotherapy, O2matic, respiratory failure, exercise, long-term oxygen treatment

## Abstract

The need for oxygen increases with activity in patients with COPD and on long-term oxygen treatment (LTOT), leading to periods of hypoxemia, which may influence the patient’s performance. This study aimed to evaluate the effect of automated oxygen titration compared to usual fixed-dose oxygen treatment during walking on dyspnea and endurance in patients with COPD and on LTOT. In a double-blinded randomised crossover trial, 33 patients were assigned to use either automated oxygen titration or the usual fixed-dose in a random order in two walking tests. A closed-loop device, O2matic delivered a variable oxygen dose set with a target saturation of 90–94%. The patients had a home oxygen flow of (mean ± SD) 1.6 ± 0.9 L/min. At the last corresponding isotime in the endurance shuttle walk test, the patients reported dyspnea equal to median (IQR) 4 (3–6) when using automated oxygen titration and 8 (5–9) when using fixed doses, *p* < 0.001. The patients walked 10.9 (6.5–14.9) min with automated oxygen compared to 5.5 (3.3–7.9) min with fixed-dose, *p* < 0.001. Walking with automated oxygen titration had a statistically significant and clinically important effect on dyspnea. Furthermore, the patients walked for a 98% longer time when hypoxemia was reduced with a more well-matched, personalised oxygen treatment.

## 1. Introduction

Dyspnea is the most debilitating symptom experienced by patients with Chronic Obstructive Pulmonary Disease (COPD) [1]. Pulmonary rehabilitation is effective in relieving dyspnea, and exercise training is the central component in any rehabilitation program [2]. Patients with advanced COPD often develop respiratory failure with chronic hypoxemia and the need for long-term oxygen treatment (LTOT) [1,3]. LTOT, which improves pulmonary hemodynamics and survival, is recommended for 15–24 h a day and the given oxygen flow rate should maintain the partial pressure of oxygen (PaO_2_) > 8 kPa at rest [3,4]. However, the oxygen requirement increases during exercise, so even though the patients receive the amount of oxygen required at rest, they still desaturate during activity, and are thereby limited by hypoxemia and dyspnea [5]. Ambulatory oxygen therapy slightly increases walking distance in patients on LTOT compared with room air, and it is recommended to use supplementary oxygen during exercise in a pulmonary rehabilitation program for patients already on LTOT [4]. However, oxygen during exercise is handled differently between countries [6,7], and this could possibly be due to the lack of specific recommendations [8].

Recently, devices have been developed that automatically adjust the oxygen flow to the dosage necessary for keeping the saturation at the recommended level. The devices are small, portable, and use feedback from the continuous measurement of the saturation to control the amount of oxygen delivered. This automated oxygen delivery has been proven to be safe and to keep patients with acute exacerbation in COPD in the acceptable saturation range for a longer period [9,10]. Two studies also revealed that automated oxygen titration reduced the time with hypoxemia during walking [11,12]. The study, by Vivodtzev et al., found that walking endurance was increased with automated oxygen compared to fixed-dose [11], while the study by Lellouche et al. did not find a significant improvement in endurance when a variable oxygen dose was compared to fixed-dose oxygen [12]. The British Thoracic Society recommends regulating oxygen flow during exercise [3]. In clinical practice, it is difficult to establish the most optimal oxygen flow as, for instance, resistance training demands a lower flow than interval training [13]. Furthermore, the clinical impact of optimal oxygen dosing on patients with hypoxemia is unknown.

Walking is an important factor for an independent social life, and field walking tests are used for assessing walking capacity. They are conducted in pulmonary rehabilitation to establish exercise intensity, limitations to exercise, and to show the effect of the intervention [14]. The six-minute walk test (6MWT) is frequently referred to in order to evaluate the immediate response of oxygen in patients with COPD [15,16]. However, studies have suggested that the endurance shuttle walk test (ESWT) would be more sensitive to a change in walking capacity, and especially when evaluating dyspnea, a standardised level of exertion should be used [14,17].

Our hypothesis was that using automated oxygen titration during walking could keep the patient’s saturation at an acceptable level for a longer period compared with usual oxygen dose, increase their endurance, and decrease their degree of dyspnea. Therefore, the purpose of this study was to evaluate the effect of automated oxygen titration compared to usual fixed-dose oxygen treatment during walking on endurance and dyspnea in patients with COPD on LTOT.

## 2. Materials and Methods

### 2.1. Patients and Study Design

The study included patients with COPD on home oxygen, and was conducted as a multicenter, double-blinded randomised crossover trial with two arms. The patients were eligible for the study if they received LTOT based on the international criteria (hypoxemic at rest without oxygen supplement, saturation < 88% and PaO_2_ < 7.3 kPa (55 mm Hg)), were able to walk at least 70 m, used ambulatory oxygen when leaving home, and cognitively were able to participate. The patients were excluded if they had conditions other than COPD limiting exercise performance or contributing to dyspnea, or had an exacerbation in COPD treated with either antibiotics or prednisolone within the preceding three weeks. Eligible patients were recruited by nurses specialised in oxygen treatment from the pulmonary section of three hospitals, Bornholm, Amager, and Hvidovre, in the capital region of Copenhagen, Denmark, over the period from 28 October 2019 to 1 December 2020.

The included patients provided written informed consent before participation, and the study was approved by the Danish Data Protection Agency and the National Committee on Health Research Ethics (H-19044780). The study was registered at ClinicalTrials.gov (NCT04123730), and the description follows the CONSORT statement for randomised crossover trials.

### 2.2. Oxygen Equipment and Randomisation

**Figure 1:**
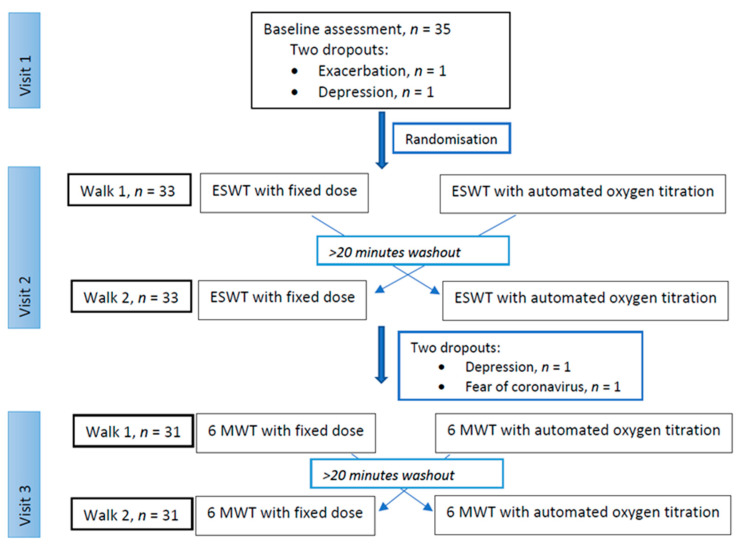
Consort flow diagram showing numbers of participants at each visit. ESWT: endurance shuttle walk test, 6MWT: six-minute walk test.

The study consisted of three hospital visits on three different days separated by more than 24 h: an enrolment visit and two test visits. On each test day, two different walks, the ESWT or the 6MWT, were performed with both the usual fixed oxygen dose and with a variable oxygen dose, automatically titrated (Figure 1). The patients performed both walks in each test on the same day to avoid dropouts and to minimise any other day-to-day fluctuation on performance.

The device used for automated oxygen titration was a closed-loop system, O2matic (O2matic Ltd, Herlev, Denmark). O2matic delivered a variable flow based on the continuous, non-invasive measurement of saturation using a pulse oximetry on a finger. Oxygen flow was adjusted to maintain the blood oxygen saturation (SpO_2_) within a target interval that was set at 90–94%, and an oxygen flow of 0–15 litres/minutes (L/min). If the saturation fell below 90%, oxygen flow increased, and if the saturation increased to above 91%, oxygen flow decreased in order to stay on target. O2matic was set in “walking mode”; in this mode, adjustments in flow were larger in response to deviations in saturation than if O2matic was used for patients at rest. The adjustments were made by O2matic every second based on average saturation for the latest 15 s. In the control arm, the fixed-dose oxygen was set at the same dosage as prescribed for the patients before entering the study (a flow rate that secured PaO_2_ ≥8 kPa at rest). O2matic was used to monitor and deliver the oxygen flow in both arms, but only in the intervention arm did O2matic adjust the oxygen flow based on the input from the pulse oximeter. All patients used a rollator as a walking aid. The medicinal oxygen cylinder and O2matic were placed in the rollator.

The order of the oxygen supply was randomised before the first test day with an allocation ratio of 1:1 using REDCap electronic data capture tools (REDCap Consortium, Vanderbilt University Medical Centre, Nashville, TN, USA) hosted at Copenhagen University Hospital, Hvidovre [18]. An independent physiotherapist prepared the oxygen setup according to a computer-generated randomisation, with O2matic in “manual mode” for fixed-dose (FD) and in “automatic mode” for automatic titration (AOT). Both the ESWT at visit two and the 6MWT at visit three were individually randomised to one of the following:

(a) visit 2: ESWT with AOT followed by FD; visit 3: 6MWT with AOT followed by FD, or
(b) visit 2: ESWT with FD followed by AOT; visit 3: 6MWT with AOT followed by FD, or
(c) visit 2: ESWT with AOT followed by FD; visit 3: 6MWT with FD followed by AOT, or
(d) visit 2: ESWT with FD followed by AOT; visit 3: 6MWT with FD followed by AOT.

The patients and the assessor (L.M.K) were blinded to the oxygen supply by covering up O2matic with a cloth during the tests.

### 2.3. Study Visits

At visit 1, demographic data were collected and the patients performed an externally paced maximal exercise test, the incremental shuttle walk test (ISWT), using their usual prescribed oxygen dose. The walking speed in the ISWT was increased until the patients had reached their maximum performance and were no longer able to continue. At the same visit, a 6MWT was performed for familiarisation according to guidelines [14].

At visit 2, the patients conducted an ESWT on a 10 m course twice and in a randomised order, without the usual two-minute warmup to avoid exhaustion too fast. The walking speed was calculated from the performance on the ISWT and was set corresponding to 75% of the estimated peak oxygen consumption. The expectation was that some of these severely ill patients would not be able to walk for a whole minute at a higher pace, and as the intension was to keep the patients walking for more than 1–3 min, the walking speed was set at 75% of the estimated peak oxygen consumption and not the usual 85%. The time walked in minutes and seconds was registered. One ESWT was performed using O2matic to deliver a variable oxygen dosage set at the chosen saturation target. The other ESWT was performed using the usual fixed-dose oxygen. In both arms, O2matic monitored pulse rate and saturation continuously during the test. The patients were given a rest of at least 20 min between tests, as recommended by the BTS guideline for home oxygen use [3]. Both saturation and dyspnea level returned to baseline levels before test two, and rollator and oxygen equipment were the same in the two tests. Every minute during the test, the patients were asked to rate their intensity of dyspnea using the modified Borg dyspnea scale CR10 with the same phrase: “From 0–10, how much difficulty is your breathing causing you right now?” [19] The patients walked for as long as possible until they were unable to continue and were asked to report the primary reason for not being able to walk any longer: dyspnea, leg fatigue/pain, exhaustion, or other reasons.

At visit 3, the patients conducted the 6MWT twice in the same way as mentioned above with fixed-dose and automated oxygen titration in random order. The 6MWT was performed with standardised instructions as a self-paced test of endurance walking capacity [14]. The walked distance in metres was recorded. Before and after completing the 6MWT, the patients rated the intensity of their dyspnea.

### 2.4. Outcome Measures

The primary outcome was a difference in experienced dyspnea in the ESWT at isotime between automated and fixed-dose oxygen delivery. Isotime was defined as the highest equivalent exercise minute achieved during each test performed by the given patient.

Secondary outcomes were:

- Difference in walking time between arms in ESWT;
- Difference in experienced dyspnea and walking distance in the 6MWT;
- Difference in average oxygen flow (L/min) between arms in both ESWT and 6MWT;
- Time spent within acceptable saturation interval (SpO_2_ 90–94%), and time spent with moderate hypoxemia (SpO_2_ < 88%) and severe hypoxemia (SpO_2_ < 85%) in both tests.

### 2.5. Statistics

The sample size was determined based on the primary outcome, the BORG CR10 dyspnea scale. The minimal clinically important difference (MCID) is 1 unit in the Borg dyspnea scale [20], and the standard deviation is expected to be 2 units. Based on an alfa of 0.05 and a power of 80%, a power analysis estimated that a sample of 33 patients was needed to examine if the intervention resulted in improvement. It was estimated that 40 patients were needed due to an expected dropout rate of 15%. Continuous variables were examined for normality using histogram and the Shapiro–Wilk test, and analysed with either a paired *t*-test (in case of normality) or the Wilcoxon signed-rank test (in case of non-normality). The test for carryover effect was performed by comparing the first and the second walk in both the ESWT and the 6MWT using the Wilcoxon signed-rank test and paired *t*-test, as appropriate. In the 6MWT, the MCID is 26 m in patients with severe COPD [15]. IBM SPSS *Statistics for Windows*, version 25.0, Armonk, NY, USA, was used for all statistical analyses.

## 3. Results

Thirty-five patients were included in the study, but two patients dropped out before randomisation (Figure 1). Baseline characteristics are presented in Table 1.

### 3.1. Endurance Shuttle Walk Test

Dyspnea, which was the primary outcome, was statistically significant reduced by a median (interquartile range) of 3 (1–4) units in the Borg score at isotime in the ESWT when walking with automated oxygen titration compared to fixed-dose, *p* < 0.001 (Table 2). The last corresponding isotime in the two arms was median 5 (3–7.5) minutes, but with significantly longer walking time in the automated oxygen arm, *p* < 0.001 (Table 2).

Dyspnea was the main reason for stopping the walk in 20 and 27 patients when using automated oxygen and fixed-dose, respectively. Four patients stopped due to leg pain in the automated, and three patients in the fixed-dose arm. Eight patients reported exhaustion as the primary reason for stopping in the automated oxygen titration group, and two patients in the fixed-dose arm.

**Figure 2:**
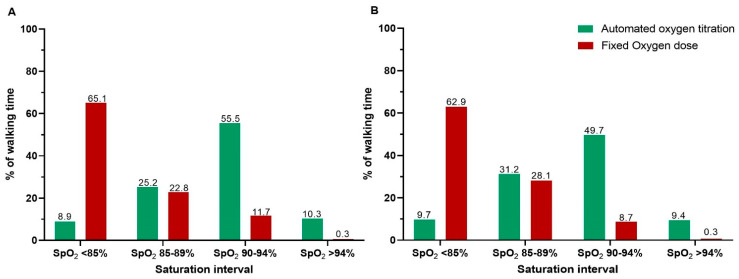
Percentage of time spent in the different oxygen saturation intervals (SpO_2_) when walking with automated oxygen titration and fixed oxygen dose in (**A**) the ESWT and (**B**) the 6MWT. The exact percentage of time in the respective interval is added on top of each bar. ESWT: endurance shuttle walk test, 6MWT: six-minute walk test.

**Figure 3:**
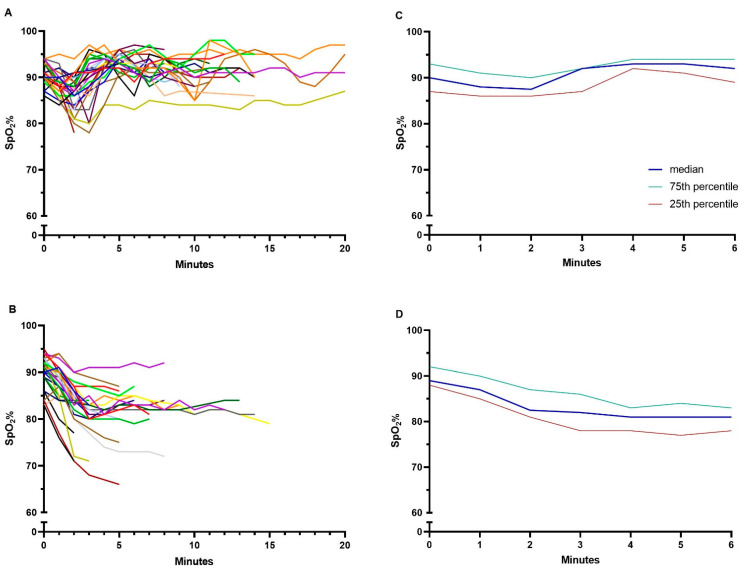
Oxygen saturation (SpO_2_) during (**A**) the ESWT using automated oxygen titration, (**B**) the ESWT using the usual fixed oxygen dose, (**C**) the 6MWT using automated oxygen titration, and (**D**) the 6MWT when using fixed oxygen dose. Data in panel (**A**,**B**) are presented as individual patients, represented with different colors, to visualise the individual walking time, and in panel (**C**,**D**) as median with 25–75% quartiles. ESWT: endurance shuttle walk test, 6MWT: six-minute walk test.

A statistically significant difference was seen between arms in saturation in favour of automated oxygen titration (Table 2, Figure 2A and Figure 3A,B). The oxygen flow increased with automated titration and exceeded 10 L/min in 27% of the patients. In the analysis testing for carryover effect, no statistical difference was seen in walking time between walk 1 and walk 2, p = 0.3.

### 3.2. The Six-Minute Walk Test

Thirty-one patients performed the 6MWT with a statistically significant difference of, median 2 (1–2) units in dyspnea, p < 0.001, and a walking difference of, mean ± SD 20.3 ± 21.7 m, both in favour of automated oxygen titration, p < 0.001 (Table 2). Eleven (35%) patients exceeded the MCID of 26 m improvement when walking with automated oxygen compared to fixed-dose, and 24 (77%) exceeded the MCID of 1 unit improvement in the Borg scale. In total, 26 (84%) patients showed a statistically significant and clinically relevant improvement in either the walking distance or in the Borg dyspnea score when receiving automated oxygen. A significant difference was seen between arms in saturation in favour of automated oxygen titration (Table 2, Figure 2B and Figure 3C,D). When testing for carryover effect no statistical difference was seen in the walking distance between walk 1 and walk 2, p = 0.08. No harms were registered during or after the tests.

## 4. Discussion

In this randomised crossover study, firstly, we found that walking with automated oxygen titration had a statistically significant and clinically important effect on dyspnea in patients with advanced COPD on LTOT. Secondly, walking time was improved by 98%, and thirdly, the oxygen demand was more well-matched when walking with automated oxygen titration using O2matic compared to the usual fixed-dose oxygen. The results support our hypotheses and suggest that oxygen therapy tailored to individual needs with the aim of preventing hypoxemia during walking can have profound relevance for patients on LTOT.

It is well known that hypoxemia, among other factors, such as dynamic hyperinflation and an increased work of breathing, leads to dyspnea [21]. O’Donnell et al. revealed in a study that breathing a high oxygen concentration can delay the ventilatory constraints observed in hypoxemic patients [22]. Even though a fixed high oxygen dose was compared with room air, the mechanisms observed in the study could potentially explain the clinical effects observed in our study. The previous study demonstrated decreased ventilatory demand and reduced dynamic hyperinflation, which resulted in an alleviation in dyspnea and improved exercise capacity [22]. Only one prior study by Vivodtzev et al. has examined the use of automated oxygen titration compared to a fixed-dose oxygen on walking endurance in patients with COPD eligible for LTOT [11]. Similar to our results, the patients received a higher oxygen flow according to their oxygen demand, and thereby improved the saturation. The study, involving 12 patients, reported a 33% improvement in endurance with an increase in delivered oxygen flow from 3.1 L/min (prescribed oxygen dose + 1 L/min) to 5.4 L/min, whereas we found an increase in endurance of 98% and a larger increase in oxygen flow from 1.6 L/min (prescribed oxygen dose) to 7.9 L/min. The larger improvement in endurance in our study, and the associated larger increase in oxygen flow, could be due to the differences regarding the response to the rapid change in SpO2 between the two devices (FreeO_2_ and O2matic), or due to how the patients in our study walked at 75% of the estimated peak oxygen consumption compared to 85% in the study by Vivodtzev et al., as a walking speed at 85% could impose other limitations on endurance than oxygen delivery.

Despite statistical significance, the average of 20 m improvement in the 6MWT in our study did not reach the MCID of 26 m, but the patients did report a relief in dyspnea. The 6MWT is not endorsed for examining dyspnea because a possible improvement in breathing could lead to an increased walking speed or less dyspnea so that the overall effect is drowned out by the combined improvement in the two parameters [17]. A standardised test with a fixed speed at isotime, such as the ESWT, is suggested to detect a possible effect on dyspnea of a given intervention [14,17]. In our study, some patients used the “extra air” to walk faster in the 6MWT; others walked at a similar speed, but at the end of the test, they reported less dyspnea. It is remarkable that 84% of the patients in our study had a clinically relevant effect of the automated oxygen titration in the 6MWT when including dyspnea as an important outcome parameter. The ESWT was more sensitive to detecting an immediate response to oxygen in our study.

Studies have shown that patients with COPD who are receiving ambulatory oxygen, and therefore carry a portable oxygen device, experience embarrassment, stigma, fear of cylinders running out of oxygen, reduced ability to travel, noise nuisance, etc. [4]. Taking this burden of ambulatory oxygen into consideration and the small effect of oxygen during activity, both the British Thoracic Society and American Thoracic Society conditionally recommend the prescription of ambulatory oxygen to patients with COPD and hypoxemia, with the main reason being to increase the time spent with LTOT during the day [3,4]. A possible explanation for the small effect of oxygen during activity could be that the patients, in general, receive an insufficient amount of oxygen during activity. We detected an average desaturation to 82.5% during walking with the usual oxygen dose, similar to what has been reported in several other studies (desaturation during walking with usual oxygen dose ranges from 80–84%) [11,23,24,25]. O2matic delivered 7.9 L/min on average to maintain the saturation in the targeted range of 90–94% during activity, but also frequently delivered a very high oxygen flow (>10 L/min). All in all, we observed that, for over 60% of the time, the patients suffered from severe hypoxemia during walking with the usual fixed-dose oxygen (Figure 2), and that the amount of oxygen required to minimise hypoxemia was higher than we expected. Furthermore, the benefits of minimising hypoxemia seem greater than we anticipated. Automated oxygen with a fast flow titration based on the change in saturation is a very promising way to rethink oxygen supplement, offering the possibility of personalised treatment for patients with LTOT, which may lead to greater benefits for the patients.

The patients and the assessor were blinded to the type of oxygen supplement, but one of the limitations of our study was that we could not rule out that, in some cases, the noise, due to the high amount of oxygen given in the nasal cannula, could have revealed the intervention. However, the tests were carried out with the same instructions to walk as far as possible; the patients were not verbally paced, and at each minute, they were asked how they experienced dyspnea. The patients were not told how long they had walked, and they were not informed about the primary outcome.

A walking test performed on patients with COPD is often stopped for safety reasons if the patients desaturate below 80% [14]. The assessor in our study conducted the tests blindly and was, therefore, unaware of a possible desaturation. However, in everyday life, some patients with COPD also experience desaturations when performing activities of daily living, for example, washing, eating, or walking [26].

In approximately 10% of the time walking with variable oxygen dose, the saturation was too high (Figure 2). When the flow exceeds 10 L/min, it can be quite uncomfortable for the patients to keep the nasal cannula in the nose, and some patients complained of a burning sensation in the nose. This could probably have been reduced by using a high flow nasal cannula. Besides patient comfort, too much oxygen can increase the risk of hypercapnia. It is a limitation that we did not take an arterial blood gas test to examine this, however a previous study did not find a worsening in hypercapnia when using variable oxygen flows in patients during walking [11]. Additionally, the high oxygen concentration was given only for a short period while monitoring the saturation and keeping this in the target, thus it imposed minimal risk for the patients.

The fast “walking mode” in O2matic, where the device responds with faster changes in flow to changes in saturation, could make the device suitable to use in an exercise setting, such as in pulmonary rehabilitation. Sufficient oxygenation could lead to improved performance during rehabilitation with less dyspnea and, therefore, greater benefits of a rehabilitation program for patients on LTOT. Further studies are needed in this field, and especially with measurements of CO_2_ levels after, for example, one hour of exercising with a high oxygen flow, to investigate the risk of hypercapnia when using O2matic. Future studies could also examine if automated oxygen titration could improve the performance of activities of daily living. However, at the moment, it is only possible to deliver high oxygen flow using hospital equipment, as the home oxygen devices are often limited to a lower flow.

In conclusion, our results provide compelling evidence for using individualised automated oxygen titration to decrease dyspnea during walking and increase walking endurance in patients with COPD on LTOT.

## Figures and Tables

**Table 1:** Characteristics of the included patients, *n* = 33.

Variables	
Men	12 (36%)
Women	21 (64%)
Age, years	72.7 ± 6.5
Body Mass Index, kg/m^2^	25.6 (21.5–29.0)
MRC	4 (3–4)
CAT, points 0–40	17.3 ± 6.2
LTOT dose, L/min	1.6 ± 0.9
SpO_2_ at rest with LTOT, %	91.7 ± 2.3
Borg CR10 dyspnea at rest	1 (0–1.5)
Hospitalisations last year, no.	1.0 (0–2.0)
Smocking status, no.	
• Smoker	1 (3%)
• Ex-smoker	31 (94%)
• Never-smoker	1 (3%)
Pack years	44.5 (30.7–60.0)
Hand grip strength, men, kg	37.2 ± 9.8
Hand grip strength, women, kg	21.3 ± 5.7
ISWT, meters	120 (85–165)
Borg CR 10 after ISWT	7 (5–8)
ESWT, walking speed, km/t	2.44 (2.09–2.72)
Usual walking aid, no.	
• No walking aid	12 (36%)
• Rollator	19 (58%)
• A walking stick	2 (6%)
FEV_1_, liter	0.6 (0.5–0.9)
FEV_1_, % of predicted	28 (23–41)
FEV_1_/FVC, ratio	0.43 (0.35–0.48)
Comorbidities, no.	
• Ischemic heart disease	4 (12%)
• Heart failure	8 (24%)
• Diabetes	1 (3%)
• Osteoporosis	18 (55%)
• Osteoarthritis (hip or knee)	10 (30%)
• No comorbidities	6 (18%)
• ≥2 comorbidities	13 (39%)

Data are presented as mean ± standard deviation (SD), median with interquartile range (IQR) or counted number with percentage (%). MRC: Medical Research Council Dyspnea Scale; CAT: COPD assessment test; LTOT: long-term oxygen therapy; SpO_2_: peripheral oxygen saturation; ISWT: incremental shuttle walk test; ESWT: endurance shuttle walk test; FEV_1_: forced expiratory value in the first second; FVC: forced vital capacity.

**Table 2:** Differences in outcomes between arms.

**Endurance Shuttle Walk Test, *n* = 33**			
**Variables**	**Fixed-Dose**	**Automated Dose**	***p*-Value**
Dyspnea, Borg CR10 score	8 (5–9)	4 (3–6)	<0.001
Walking time, minutes	5.5 (3.3–7.9)	10.9 (6.5–14.9)	<0.001
Time with target saturation, minutes	0.7 (0–1.1)	5.3 (2.6–7.8)	<0.001
Median saturation, %	82 (82–84)	91 (90–94)	<0.001
Average flow, L/min	1.6 ± 0.9	7.9 ± 3.1	<0.001
**Six-Minute Walk Test, *n* = 31**			
**Variables**	**Fixed-Dose**	**Automated Dose**	***p*-Value**
Dyspnea, Borg CR10 score	7 (6–8)	5 (3–7)	<0.001
Walking distance, meters	271.3 ± 65.1	291.6 ± 67.4	<0.001
Time with target saturation, minutes	0.3 (0–1.0)	3.0 (1.5–3.9)	<0.001
Median saturation, %	81 (78–85)	92 (88–93)	<0.001
Average flow, L/min	1.6 ± 0.9	8.1 ± 3.6	<0.001

Data presented as median (IQR) or mean ± SD. Primary and secondary outcome measurements when walking with the fixed oxygen dose and the automated oxygen titration. Borg dyspnea scale CR10 given at isotime. Target saturation: 90–94%.

## Data Availability

The data presented in this study are available on request from the corresponding author. The individual data are not publicly available due to privacy of the participating patients.
